# Cutaneous Myiasis in a Healthy Young Adult From Saudi Arabia: A Report of a Rare Case

**DOI:** 10.7759/cureus.72790

**Published:** 2024-10-31

**Authors:** Abdulrahman Saleh Aldairi, Yusra Bundagji, Faris Alsaedi, Reda Saifaldeen, Ethar Alsaedi, Moroj Mohammed Alzahrani, Homaid Alotaibi

**Affiliations:** 1 Department of Dermatology, King Faisal Hospital, Ministry of Health, Makkah, SAU; 2 Department of Medicine and Surgery, Umm Al-Qura University, Makkah, SAU

**Keywords:** central punctum, cutaneous myiasis, furuncular myiasis, larvae, myiasis

## Abstract

Myiasis is a parasitic infection of the skin tissue caused by larvae, which are commonly known as maggots, that is typically observed in the tropical and subtropical areas of Africa and the Americas. Cutaneous myiasis is the most prevalent form of myiasis and is categorized as furuncular, creeping (migratory), or wound (traumatic) myiasis based on its clinical presentation. Few cases of cutaneous myiasis have been observed in Saudi Arabia, and most of these have been observed in southern Saudi Arabia. We present the case of a 14-year-old female patient in Makkah, Saudi Arabia, who developed several itchy, painful, and oozing skin lesions after spending one month in a rural area of Jizan. The patient exhibited multiple raised erythematous boil-like lesions with a central punctum, and a foreign body protruded from one of the lesions. The foreign body was manually removed from the lesion using forceps. Furuncular myiasis was diagnosed because the foreign body comprised larvae. Subsequently, manual removal of all larvae was performed by applying pressure and using forceps. A course of oral antibiotics was administered to treat the bacterial infection, which developed as a complication of a preexisting parasitic infection. The patient was discharged after full recovery. Physicians should be aware of such cases because they are relatively rare in Saudi Arabia. To prevent misdiagnoses, careful medical history and examination should be performed.

## Introduction

Myiasis is a parasitic infection caused by larvae, frequently referred to as maggots, comprising a variety of dipterous (two-wings) flies [1-3]. The term “myiasis” originates from the Greek word myia, which means “fly” [3]. Such infections can be classified based on the anatomical location or the relationship between the parasite and the host. The anatomically based classification considers the affected region, such as the cutaneous, oral, nasopharyngeal, ophthalmic, gastrointestinal, urogenital, and auricular regions [4]. The relationship-based classification considers parasite-host interactions (ecologically), including specific (obligatory), semi-specific (facultative), and accidental (pseudomyiasis) interactions [4]. Cutaneous myiasis is the most frequently encountered skin disease and the fourth most common travel-associated skin disease. Cutaneous myiasis can be further categorized according to its clinical presentation as furuncular, creeping (migratory), or wound (traumatic) myiasis [5]. These infections are frequently observed in domestic and wild mammals worldwide [2,3]. The larvae associated with these infections make use of the host’s dead or live tissues, body fluids, or ingested food [6]. A poor socioeconomic status, poor personal hygiene, chronic skin diseases, advanced age, and young age are possible risk factors for cutaneous myiasis in humans [6]. We describe a case of cutaneous myiasis confirmed by larvae extraction in a healthy young Saudi girl in Makkah City.

## Case presentation

**Figure 1:**
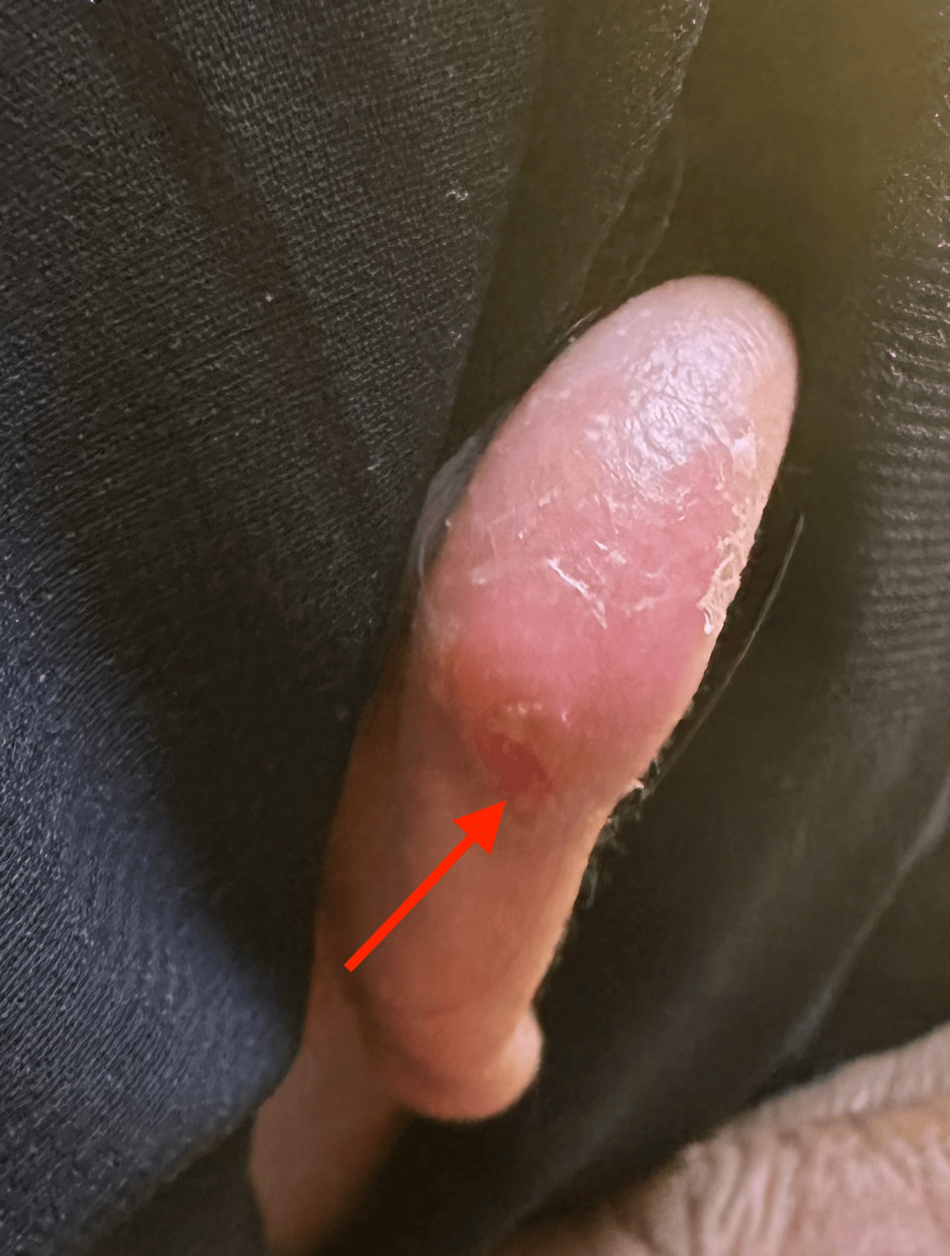
Primary skin lesion associated with furuncular myiasis located on the helix of the right ear and characterized by prominent erythema and a central punctum (arrow).

**Figure 2:**
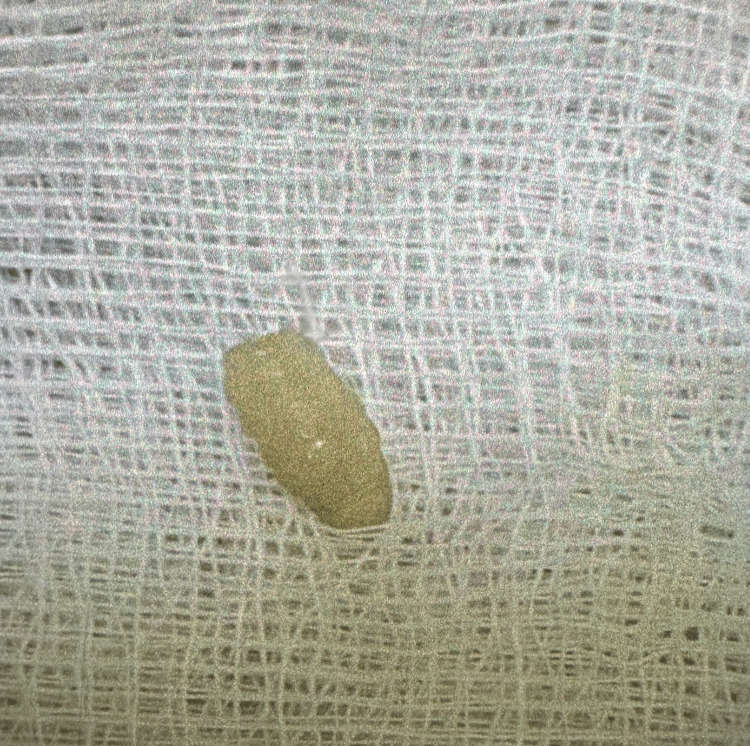
Larva extracted from the helix of the right ear.

**Figure 3:**
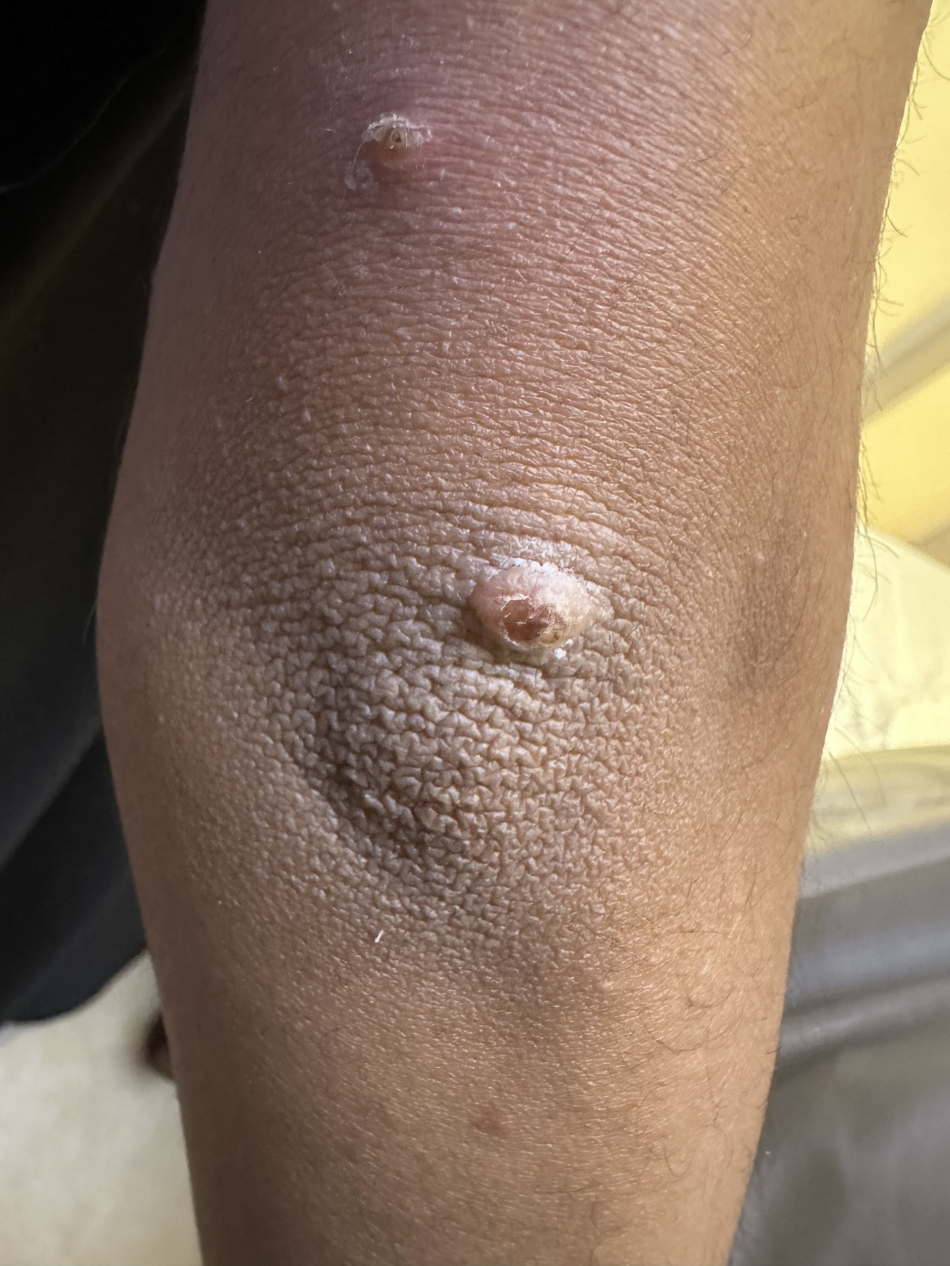
Two raised erythematous lesions located on the left upper extremity with a central punctum consistent with furuncular myiasis.

**Figure 4:**
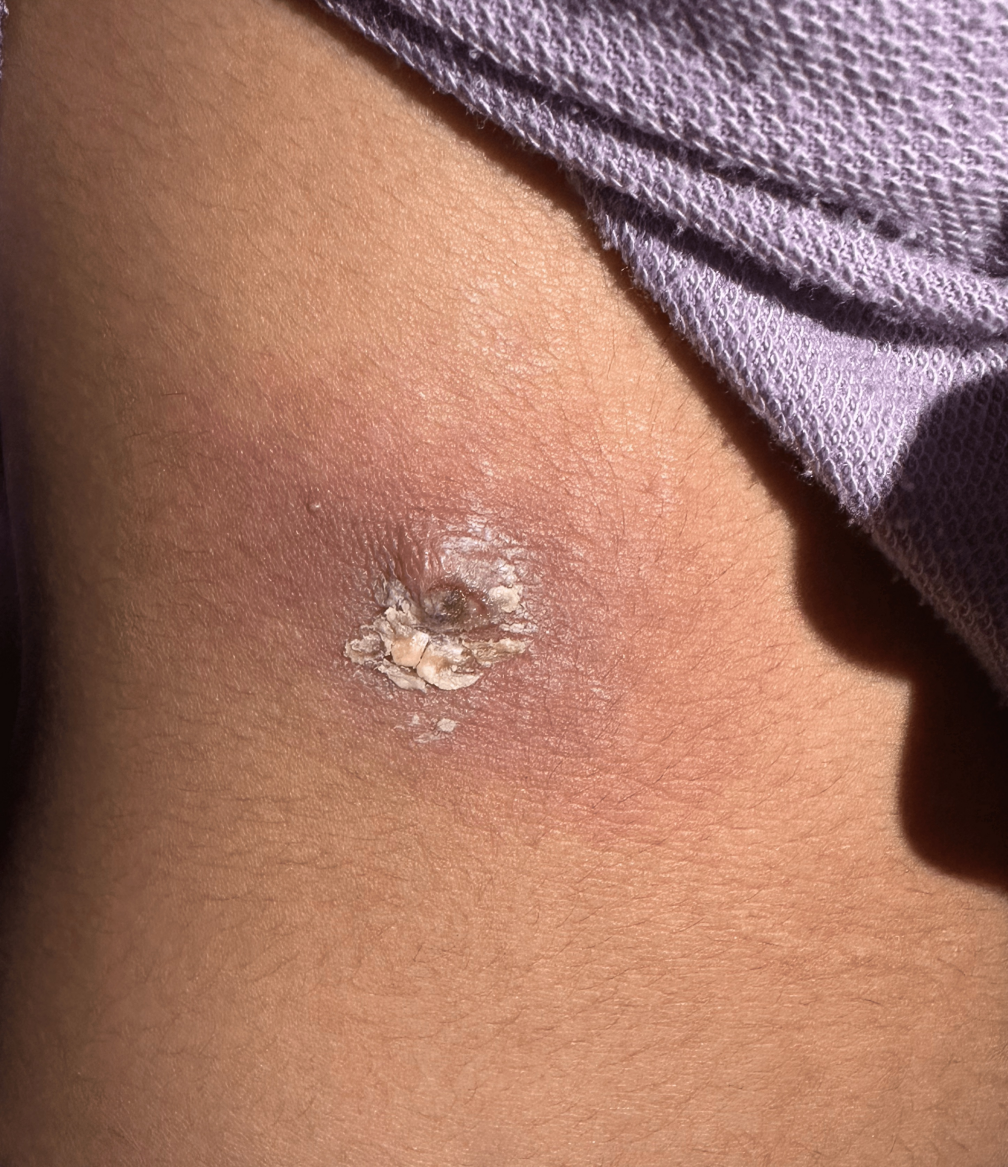
A skin lesion (furuncular myiasis) on the abdomen featuring a central punctum with a well-defined border and surrounded by yellowish crust and erythema.

A 14-year-old Saudi girl living in Makkah City presented to the emergency department with her parents because she had developed several itchy, painful skin lesions over the past five days. The family had recently spent one month in a rural area of Jizan, which is a city located in the southern region of Saudi Arabia. Additionally, the patient reported that she sustained multiple mosquito bites during the days before she returned to Makkah. The first skin lesion was a small, red papule on the outer surface of the right ear. Two days later, the patient observed more lesions on various areas of her body, including the left arm, forearm, and abdomen. These lesions became larger and had a central punctum with visible discharge; however, they were not associated with fever. A review of the systems did not reveal any remarkable findings, and she had no family history of similar symptoms. Additionally, she denied having any contact with ill individuals or animals. During the physical examination, we noticed part of the larval body protruding from the helix of her right ear (Figure 1); therefore, we gently removed it using forceps (Figure 2). The patient had multiple red, raised, boil-like lesions with a central punctum spread across her left upper extremity (Figure 3) and one lesion on her abdomen (Figure 4).

Based on the clinical observations of the extracted larvae, furuncular myiasis was diagnosed. A complete blood count (CBC) revealed normal results. Six maggots were extracted using forceps after applying gentle pressure around each lesion. Because of the long history of the lesions, a swab of the lesion on the helix of the right ear was performed to obtain a sample for any secondary bacterial infection potentially caused by wound contamination. At the time of discharge from the emergency department, fusidic acid 2% ointment was prescribed for five days. During a follow-up appointment at the dermatology clinic one week later, the culture results of the sample obtained with the swab revealed Staphylococcus aureus. Consequently, we prescribed a course of amoxicillin-clavulanic acid 500 mg/125 mg, administered orally three times daily for seven days. The patient and her parents were satisfied with the treatment results. Marked improvement of all previously treated skin lesions and normal general examination results were observed. 

## Discussion

Myiasis is considered a rare condition in humans; however, it is regularly observed in tropical and subtropical areas of Africa and the Americas [7]. Furthermore, flies prefer warm, humid conditions, which are prevalent during the summer season in regions with temperate climates [8]. *Dermatobia hominis* and *Cordylobia anthropophaga* are the primary causes of furuncular myiasis [9]. *D. hominis*, which is commonly referred to as the human or tropical botfly, is the predominant cause of myiasis in tropical regions such as Mexico, South America, Central America, and Trinidad [10]. In contrast, *C. anthropophaga*, also known as the tumbu fly, is native to sub-Saharan Africa [10]. The categorization of myiasis based on the parasite-specific location of the host, known as the anatomical classification, is useful for practical diagnoses. The anatomical classification system was initially proposed by Bishopp in 1921 [11], and it was later updated by James in 1947 [12] and Zumpt in 1965 [13] (Table 1) [9].Cutaneous myiasis has been identified in two cities, Asir and Al-Baha, in Saudi Arabia [18,19]. Nevertheless, the precise number of myiasis cases remains unknown because of underdiagnosis or failure to report cases to the appropriate authorities. However, the confirmed cases in various regions of Saudi Arabia indicated that the causative agents, particularly D. hominis and C. anthropophaga, have established an endemic prevalence. A unique feature of our case was that the patient did not travel outside the borders of Saudi Arabia. Some studies have categorized the southern regions of the Arabian Peninsula as part of the Afrotropical zoogeographical belt, where C. anthropophaga is the dominant species [20]. Alotaibi et al. [20] reported a single case with a similar history of travel to the southern parts of Saudi Arabia that was caused by C. anthropophaga. Treatment options for furuncular myiasis include suffocating the larvae with petroleum jelly and then removing them [20]. The lesion was compressed using forceps so that the larvae could be extracted; thereafter, irrigation of the wound and petroleum gauze packing were performed [20]. Oral and topical ivermectin have shown efficacy in such cases [9]. Occasionally, surgical intervention is performed to explore and extract larvae from the affected area. Secondary bacterial infections can occur with wound infections or incomplete removal of the larvae during extraction, resulting in the need for antibiotics [6].

**Table 1:** Anatomical classification of myiasis Source: [9]

Bishopp classification in 1921 [11]	James classification in 1947 [12]	Zumpt classification in 1965 [13]
Bloodsucking	Bloodsucking	Sanguinivorous
Tissue-destroying	Furuncular	Dermal/subdermal
Subdermal migratory	Creeping	
	Traumatic/wound	
	Anal/vaginal	
Infestation of the head passages	Nose, mouth, sinuses	Nasopharyngeal
	Aural	
	Ocular	
Intestinal/urogenital	Enteric	Intestinal
	Anal/vaginal	
Intestinal/urogenital	Bladder, urinary passages	Urogenital
	Anal/vaginal	

The ecological classification system, which is based on parasite-host interactions, was originally used to improve the practical diagnosis and is advantageous because it allows the detection of a single species that can infest many anatomical regions as well as distinct species that can specifically target the same location (Table 2) [9].

**Table 2:** Ecological classification of myiasis Source: [9]

Ecological classification		Description
Specific (obligatory)		Parasite dependent on host for part of its life cycle.
Semi-speciﬁc/facultative	Primary	Free living and may initiate myiasis.
	Secondary	Free living and unable to initiate myiasis; may be involved once animal is infested by other species.
	Tertiary	Free living and unable to initiate myiasis; may be involved when host is near death.
Accidental/pseudomyiasis		Free-living larva and not able to complete its life cycle; causes pathological reaction when accidentally in contact with the host.

**Figure 5:**
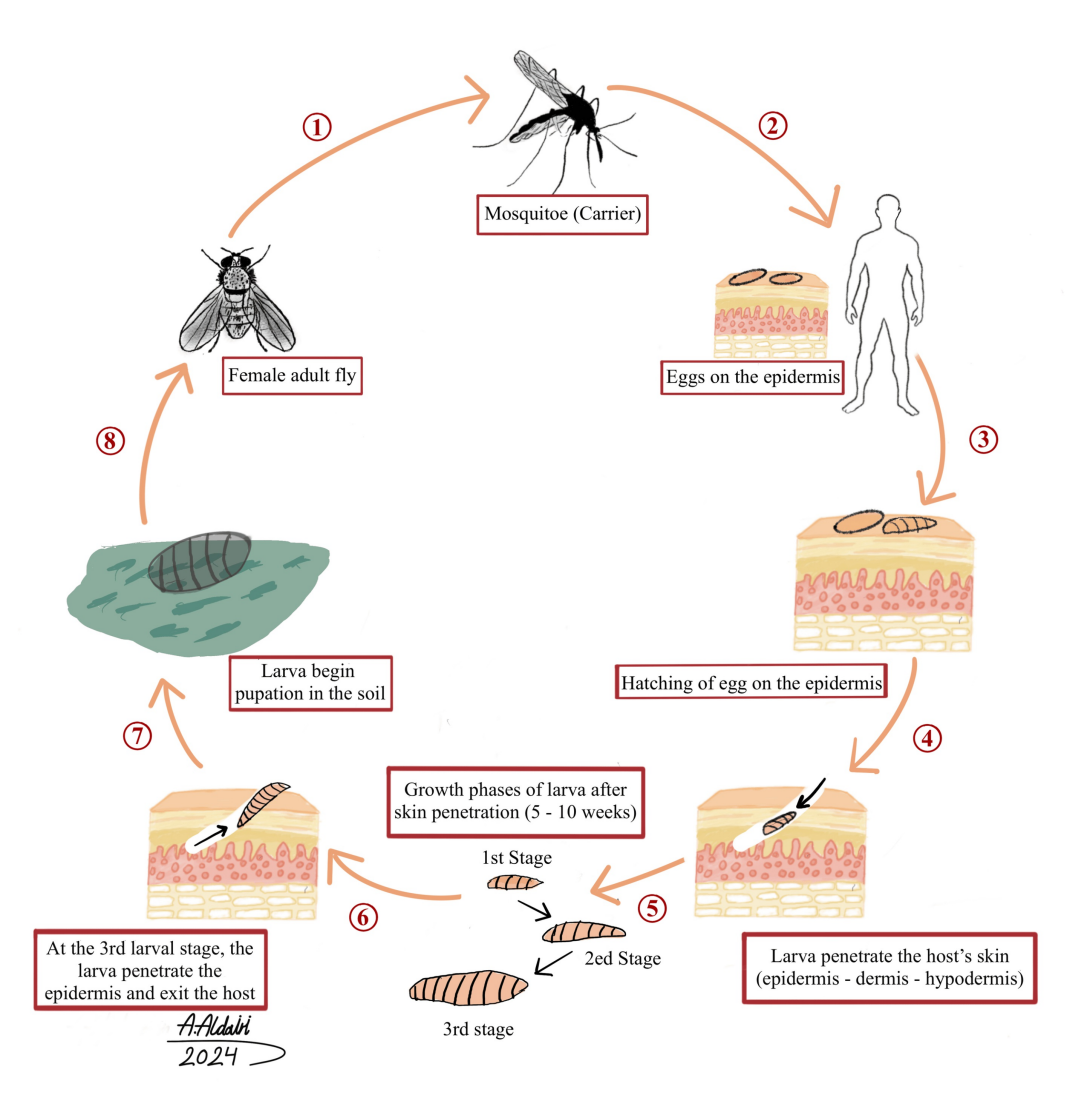
The life cycle of myiasis larvae Figure Credits: Abdulrahman Saleh Aldairi

Physicians should carefully obtain the patient’s medical history, especially when it includes a remarkable history of itchiness, stabbing or sharp pain, fever, contact with animals, insect bites, travel, the sensation of movement by a foreign body inside the lesion, socioeconomic status, personal hygiene, and chronic diseases [6,14]. A physical examination will help determine the precise shape of the lesion, ultimately narrowing the differential diagnosis. Our patient had lesions with a boil-like and erythematous appearance with a central punctum surrounded by crust, thus suggesting the exudation of purulent or serosanguineous fluid. All of these characteristics should raise the suspicion of furuncular myasis. Furuncular myiasis has various potential differential diagnoses, thus increasing the probability of misdiagnosis. The differential diagnoses include furuncles, insect bites, prurigo, pyoderma [15], inflamed cysts, tungiasis, and herpes simplex [16]. During the biological cycle of myiasis larvae (Figure 5), the adult female fly deposits eggs on the abdomen of a blood-sucking arthropod, such as the mosquito, and uses a sticky substance to adhere to those eggs. This process of delivering eggs is referred to as phoresis. Within approximately one week, when the carrier (vector) insect approaches a warm-blooded host, the increased temperature stimulates the eggs to hatch, resulting in the release of larvae that penetrate the host’s skin, through either the bite site or unharmed skin, without inducing pain. This is the first instar (stage 1). Subsequently, the larvae enter the dermal layer, reach the hypodermis (subcutaneous tissue), and form a distinctive furunculoid lesion. During the span of 5 to 10 weeks, the larvae progress from the second instar (stage 2) to the third instar (stage 3). Then, they exit the host, usually at night or during the early morning, to avoid desiccation and undergo pupation in the soil. After approximately one month, the fully developed adult fly emerges to engage in mating to repeat the biological cycle [9,17].

## Conclusions

Although cutaneous myiasis is globally recognized, it remains a relatively rare condition in Saudi Arabia. However, the increasing recognition of cases in specific regions, particularly in southern Saudi Arabia, suggests that this parasitic infection may be more prevalent than previously thought. It is essential for clinicians to consider it in their differential diagnoses, even for patients without a history of travel outside the country. This emphasizes the need for heightened clinical awareness among healthcare providers to avoid misdiagnosis and ensure timely treatment. Further studies are necessary to better understand the epidemiology of myiasis in Saudi Arabia, which could improve prevention and management strategies in endemic areas.

## References

[1] Wade N, Shahi F, Mawer D, Brown N (2019). Rare cutaneous myiasis of the face due to Lund's fly (Cordylobia rodhaini) in a British traveller returning from Uganda. BMJ Case Rep.

[2] Zaglool DA, Tayeb K, Khodari YA, Farooq MU (2013). First case report of human myiasis with Sarcophaga species in Makkah city in the wound of a diabetic patient. J Nat Sci Biol Med.

[3] Robbins K, Khachemoune A (2010). Cutaneous myiasis: a review of the common types of myiasis. Int J Dermatol.

[4] McGraw TA, Turiansky GW (2008). Cutaneous myiasis. J Am Acad Dermatol.

[5] Caumes E, Carrière J, Guermonprez G, Bricaire F, Danis M, Gentilini M (1995). Dermatoses associated with travel to tropical countries: a prospective study of the diagnosis and management of 269 patients presenting to a tropical disease unit. Clin Infect Dis.

[6] Al Juaid A, Al Zahrani W (2017). Furuncular myiasis in a child: a case report and literature review. Saudi J Med Med Sci.

[7] Crosskey RW, White GB (1977). The Afrotropical Region. J Nat Hist.

[8] Schwartz E, Gur H (2002). Dermatobia hominis myiasis: an emerging disease among travelers to the Amazon basin of Bolivia. J Travel Med.

[9] Francesconi F, Lupi O (2012). Myiasis. Clin Microbiol Rev.

[10] Davis RF, Johnston GA, Sladden MJ (2009). Recognition and management of common ectoparasitic diseases in travelers. Am J Clin Dermatol.

[11] Patton WS (1921). Notes on the myiasis-producing Diptera of man and animals. Bull Entom Res.

[12] (1947). The flies that cause myiasis in man. https://www.biodiversitylibrary.org/item/132463.

[13] Zumpt F (1965). Myiasis in Man and Animals in the Old World: A Textbook for Physicians, Veterinarians and Zoologists. https://www.cabidigitallibrary.org/doi/full/10.5555/19651000210.

[14] Magram WS, Albakri HY, Althobaity AN, Makki RM, Monaqil AT (2023). Hand furuncular myiasis of an infant in the Western Region of Saudi Arabia: a case report. Cureus.

[15] Sharma P, Pai HS, Pai GS (2008). Furuncular myiasis mimicking pyoderma. Indian J Dermatol Venereol Leprol.

[16] Boruk M, Rosenfeld RM, Alexis R (2006). Human botfly infestation presenting as peri-auricular mass. Int J Pediatr Otorhinolaryngol.

[17] Gordon PM, Hepburn NC, Williams AE, Bunney MH (1995). Cutaneous myiasis due to Dermatobia hominis: a report of six cases. Br J Dermatol.

[18] Afifi MA, Jiman-Fatani AA, Alsiny FI, Anshasi WS (2015). A new focus of autochthonous transmission of Cordylobia anthropophaga in Saudi Arabia. J Microsc Ultrastruct.

[19] Omar MS, Abdalla RE (1992). Cutaneous myiasis caused by tumbu fly larvae, Cordylobia anthropophaga, in southwestern Saudi Arabia. Trop Med Parasitol.

[20] Alotaibi S, Al Shahrani D, Alzomor O (2016). Cutaneous myiasis in Saudi infant: a rare case report. Int Med J Health.

